# A new iron supplement: The chelate of pig skin collagen peptide and Fe^2+^ can treat iron-deficiency anemia by modulating intestinal flora

**DOI:** 10.3389/fnut.2022.1055725

**Published:** 2022-12-22

**Authors:** Shan Jiang, Weichao Dong, Zhen Zhang, Jing Xu, Haoran Li, Jiayu Zhang, Long Dai, Shaoping Wang

**Affiliations:** ^1^School of Pharmacy, Binzhou Medical University, Yantai, China; ^2^Institute of Chinese Materia Medica, China Academy of Chinese Medical Sciences, Beijing, China; ^3^School of Pharmacy, Shandong University of Traditional Chinese Medicine, Jinan, China

**Keywords:** iron deficiency anemia, iron nutritional supplement, peptides-ferrous chelate, structural characterization, gut flora

## Abstract

**Introduction:**

Iron deficiency anemia (IDA) is one of the most common nutritional diseases encountered all over the world. Nowadays, oral iron supplementation is still the mainstay of IDA treatment.

**Methods:**

In this study, a new iron nutritional supplement named pig skin collagen peptides ferrous chelates (PSCP-Fe) was prepared, and its structure was characterized by the scanning electron microscopy, sykam amino acid analyzer and Fourier transform infrared spectroscopy (FTIR). The anti-IDA activity of PSCP-Fe was evaluated in low-Fe^2+^ diet-induced IDA in rats. 16S amplicon sequencing technology was then used to reveal the mechanism of PSCP-Fe against IDA.

**Results:**

The results of amino acid analysis and FTIR showed that aspartic acid (Asp), arginine (Arg), histidine (His), glutamic acid (Glu), cystine (Cys), and lysine (Lys) residued in PSCP chelated readily with Fe^2+^ through their functional groups. PSCP-Fe treated reversed the hematology-related indexes, such as red blood cells (RBC), hemoglobin (HGB), hematocrit (HCT), mean corpuscular volume (MCV), mean corpuscular hemoglobin (MCH), mean corpuscular hemoglobin concentrate (MCHC), serum ferritin (SF), serum hepcidin (HEPC) and serum transferrin receptor (TFR). And its regulatory action was better than that of FeSO_4_. Moreover, PSCP-Fe alleviated the hepatocyte apoptosis and necrosis, Fe^2+^ loss, and injury in IDA rats. In addition, PSCP-Fe could significantly retrace the disturbed profile of gut microbiota in IDA rats (*p* < 0.05) and significantly up-regulated the relative abundances of nine bacterial genus, including *Lactobacillus, Alloprevotella, unclassified_of_Oscillospiraceae*, and *NK4A214_group* (*p* < 0.05). It could also downgrade the relative abundances of *Subdoligranulum* and *Coriobacteriaceae_UCG-002* (*p* < 0.05). The results of Spearman’s correlation analysis and distance-based redundancy analysis (db-RDA) revealed that *Subdoligranulum* and *Christensenellaceae*_R-7_group may be potential microbial markers for effective PSCP-Fe action in the treatment of IDA.

**Discussion:**

Overall, our results elucidate the interactions between gut bacteria and related cytokines and reveal the mechanisms underlying the anti-IDA effect of PSCP-Fe. They will thus provide a theoretical foundation for PSCP-Fe as a new iron nutritional supplement.

## Introduction

Iron deficiency anemia (IDA) is the most common nutritional deficiency in the world (1). The World Health Organization (WHO) estimates that two billion individuals, mostly residing in low- and middle-income countries, are influenced by IDA due to a variety of causes, including parasitic infections, nutritional deficiencies, chronic diseases, hemoglobinopathies, and lead poisoning (2). Moreover, IDA is frequently identified in high-risk groups in developed countries, such as infants, women of childbearing age, and pregnant women (3, 4). Clinical research indicates that IDA is an independent risk in multiple diseases. Children with IDA often suffer from intellectual disability, non-specific immune deficiency, inflammation, and cognitive impairment. Adults suffering from IDA may face tumors, paralysis, tissue ischemia, and digestive system diseases (3). Although physiological and nutritional studies have confirmed that many factors can cause IDA, an insufficient intake of iron through diet and malabsorption of iron are predominant causes for the occurrence of IDA. Therefore, the global incidence of IDA can be reduced by increasing the intake of iron-rich foods and improving iron absorption.

Oral iron supplements are the mainstay of treatment for IDA. Iron can be supplemented as inorganic and organic salts of iron, amino acid-iron, and peptide-iron chelates (3, 4). However, inorganic iron salts have many disadvantages, such as lower iron bioavailability, lower absorption, and poor stability, which severely restrict their clinical application. In addition, inorganic iron salts can also cause colitis and liver damage. Recently, many studies have shown that bioactive peptides from animals, plants, and marine algae, exhibit numerous merits, such as low toxicities, chelation sites, and excellent bioactivities as compared to prototype proteins. These peptides can be released from their parent protein *via* gastrointestinal digestion or enzymatic hydrolysis (5). Usually, food-derived bioactive peptides, containing between 2 and 20 amino acids, can be used to prepare nutritional supplements with iron chelates. Some ferrous-peptide chelates have been reported and applied to treat IDA, such as chickpea peptide-iron chelates and ferrous-hairtail peptide chelates (6, 7).

Improvement in the microenvironment for iron to be absorbed effectively is another approach to the treatment of IDA. The gut bacteria, i.e., the “new organ of the human body,” may act as a key modulator of iron homeostasis (8). In the intestine, despite its pivotal role in cellular proliferation, free Fe^2+^ may generate toxic reactive oxygen species (ROS), which can affect intestinal integrity by inducing oxidative stress (9). In addition, unabsorbed Fe^2+^ can stimulate virulence of pathogenic bacteria (e.g., *Escherichia coli*, *Clostridium Welchii*, *Enterococcus faecalis*), which contributes to an oxidative proinflammatory environment. Gut probiotics, including *Lactobacillus*, *Bacillus*, *Bacteroides*, and *Clostridium*, can inhibit the expression of transferrin receptor (TFR), thereby reducing the proportion of Fe^2+^ in gut and blood, which reverses the above-mentioned adverse reactions (10). In addition, the gut probiotics can release multiple metabolites [e.g., branched-chain amino acids, bile acids, short-chain fatty acids (SCFAs), and nucleosides] by fermenting nutrients, which can improve intelligence and immunity by increasing the release of neurotransmitters and immune factors (11). Therefore, gut probiotics may help overcome the challenges in IDA treatment by modulating intestinal bacteria.

Pig skin, which is often used as a by-product from meat processing premises, is associated with environmental problem and low economic value. Thus, the high value-added utilization of pig skin needs to be valued and solved (12). Based on morphology and physiological functions, domestic pig skin seems to be the closest to human skin in composition (13). For this reason, it is a good model for studies on wound healing and permeability (13, 14). It is rich in collagen, and thus, is marked as a good source of active peptides. Pig skin is also used as a raw material for the preparation of new Ejiao—a blood tonic in Traditional Chinese Medicine that has been used for 2000 years. However, there are only a few studies that explore the use of pig skin collagen peptides (PSCP) for the preparation of ferrous chelates. The therapeutic effect of these chelates on IDA has never been reported, and thus, the modulation of gut microbiota in the IDA model remains ambiguous. In this study, we first prepared PSCP by enzymatic hydrolysis of pig skin with trypsin and pepsin. PSCP was then chelated with Fe^2+^ to obtain the PSCP-ferrous chelates (PSCP-Fe). The microstructure and chelation sites in PSCP-Fe were identified *via* multiple analytical instruments. An IDA rat model was then established to evaluate the potential of PSCP-Fe for the treatment of IDA. The related mechanisms of PSCP-Fe against IDA were also revealed by correlation analysis of gut microbiota structure and cytokines.

## Materials and methods

### Materials and reagents

Fresh pig skin was purchased from Jinluo Foods Co., Ltd. (Yantai, China) and was stored at −20°C until further experimentation. Pepsin (3,000 U/mg) and trypsin (1,000–1,500 U/mg) were obtained from Shanghai Lanji Technology Development Co., Ltd. (Shanghai, China). Ferrous chloride (molecular weight 126.75 Da) was purchased from Shanghai Biological Technology of Vibration Spectrum Co., Ltd. (Shanghai, China). Ferrous sulfate tables were purchased from Shanxi Lijiu Pharmaceutical Co., Ltd. (Shanxi, China). Ascorbic acid, anhydrous ethanol, and other reagents used in this study were of analytical grade. The Prussian Blue Stain Kit and hematoxylin-eosin/HE staining kit were obtained from Solarbio Technology Co., Ltd. (Beijing, China).

### Preparation and characterization of PSCP-Fe

#### Preparation of PSCP-Fe

Collagen was extracted from pig skin according to the method described by He et al. (15). After being defatted by petroleum ether, collagen was added to deionized water to obtain a concentration of 10% (weight/volume, w/v). The mixture was subjected to continuous enzymatic hydrolysis with pepsin (1.0% of the substrate, pH 1.5, 2 h) and trypsin (1.0% of the substrate, pH 8.0, 3 h) at 40°C. Finally, the enzyme solution was soaked in boiling water for 15 min to inactivate the enzymes. Samples were then centrifuged at 4,000 rpm for 10 min, and the supernatant was subjected to ultrafiltration using a 3 kDa cut-off membrane (Millipore Corporation, Bedford, MA, USA). The filtrate solution was lyophilized (−80°C, 48 h) to obtain the PSCP powder.

The PSCP-Fe was prepared following the procedure described by Ma et al. with minor adjustments (16). The PSCP (5%) were suspended in distilled water (20 mL, pH 7.0), mixed with 300 μL of FeCl_2_ (0.01%, w/v) and 75 μL of ascorbic acid (0.01%, w/v) at pH 5.0. The mixture was stirred at 40°C for 40 min, and anhydrous ethanol (6 of the solution) was added to remove unnecessary peptides. The mixture was centrifuged at 4,000 rpm for 10 min, and the precipitate, referred to as PSCP-Fe, was lyophilized (−80°C, 48 h). After the binding treatment, ferrous content in PSCP-Fe was measured by inductively coupled plasma atomic emission spectroscopy (ICP-AES, Perkin Elmer, Waltham, MA, USA) according to the method of Li et al. (17).

#### Structural characterization of PSCP-Fe

The occurrence of chelation reactions often alters the apparent structure of the peptide. Hence, the scanning electron microscopy (Phenom XL, Phenom-World BV, Eindhoven, Holland) was used to distinguish PSCP-Fe and PSCP by applying 10 mA current and 5 kV voltage. The chelating ability of peptides and Fe^2+^ is closely associated with the types of amino acid residues present. The amino acid composition of PSCP-Fe was characterized by a known method (16) with minor modifications. 100 mg of PSCP-Fe was hydrolyzed with 6 M HCl at 110°C for 24 h. The mixture was then analyzed using a Sykam amino acid analyzer (S-433D, Sykam Corporation, Munich, Germany). The chelation sites on PSCP and ferrous could be more easily diagnosed using a Fourier transform infrared spectroscopy (FTIR), whose experimental design was as follows: 5.0 mg each of PSCP and PSCP-Fe mixed with potassium bromide were flaked and then analyzed using an FTIR spectrometer (Shimadzu IRTracer-100, JPN) in the wavelength range of 4,000–400 cm^–1^ at 30°C.

### Evaluation of the effect of PSCP-Fe in the IDA rat model

#### Animals, diets, and experimental design

Forty-eight male Sprague-Dawley (SD) rats (SPF weighing 110 ± 10 g) were purchased from Jinan Pengyue Laboratory Animal Breeding Co., Ltd. (Shandong, China, SYXK (RU) 2019–0003). All rats were housed at room temperature (24 ± 2°C) with humidity (50 ± 5%) and 12 h light/12 h dark cycle. The animal usage protocol was approved by the Institutional Animal Care and Use Committee at Bin Zhou Medical University (approval certificate number: 2020–090). The animal facilities and protocols complied with the Guide for the Care and Use of Laboratory Animals [USA National Research Council, 1996 (18)].

Deionized water was provided continuously to all rats. After 7 days, all rats were randomly divided into control group (Control, 8 rats) and IDA groups (40 rats) based on body weights (BW). Rats in the control group were fed normal rodent chow (45 mg Fe kg^–1^ of diet, Pengyue, Shandong, China), and those in the IDA groups were fed a low-Fe^2+^ diet (4.93 mg Fe kg^–1^ of diet, Beijing Keao Xieli Animal Feed Co., Ltd., Beijing, China) for 5 weeks (6, 16). The health of the IDA model rats was assessed by hemoglobin (HGB), BW, and mental state of rats compare with those in the control group. Rats in the IDA groups were again allocated into five groups: the model group (Model, *n* = 8), the FeSO_4_ group (FeSO_4_, 1.62 mg Fe/100 g BW, *n* = 8), the low dose PSCP-Fe group (LPSCP-Fe, 0.81 mg Fe/100 g BW, *n* = 8), the medium dose PSCP-Fe group (MPSCP-Fe, 1.62 mg Fe/100 g BW, *n* = 8) and the high dose PSCP-Fe group (HPSCP-Fe, 3.24 mg Fe/100 g BW, *n* = 8). PSCP-Fe solutions were freshly prepared in distilled deionized water and intragastric administration was performed once per day for 3 weeks.

#### Biological sample collection and preservation

At the end of the experimental period, fresh feces of all rats were collected in sterile plastic tubes and then stored at −80°C until further analysis. The rats were fasted for 12 h before being anesthetized with 10% chloral hydrate. Blood samples from the abdominal aorta were collected and divided into two components. A component was used to analyze blood-related parameters associated with IDA, and another component was centrifuged at 4,500 rpm/min for 10 min at 4°C. Liver and colon tissues were stored in a 4% paraformaldehyde solution for histological analysis.

#### Determination of hematology-related indexes in IDA rats

The levels of red blood cells (RBC), HGB, hematocrit (HCT), mean corpuscular volume (MCV), mean corpuscular hemoglobin (MCH), and mean corpuscular hemoglobin concentrate (MCHC) were determined using an automated hematology analyzer (BC-3000, Tokyo Mairui Co., Tokyo, Japan). Serum ferritin (SF), serum hepcidin (HEPC), and TFR were measured using enzyme-linked immunosorbent assay (ELISA) kits (Shanghai Enzyme-linked Biotechnology Co., Ltd., Shanghai, China).

#### Evaluation of liver and colon damage

A long-term iron-deficient diet causes the imbalance of liver function and iron loss. In this research, hepatic tissues fixed in 4% paraformaldehyde solution were dehydrated, embedded in paraffin, and cross-sectioned into 4-μm thick slices. The slices were then treated with HCl (5%) to release ferric ions, followed by treatment with 5% potassium ferrocyanide (Sigma, MO, USA) to produce insoluble ferric ferrocyanide and counterstained with the neutral red (18, 19). In addition, the H&E staining of colon tissue was carried out according to the previous method (20). Briefly, colon tissues were cut into 5 μm-thick sections and stained with the H&E kit (Solarbio Science and Technology, Beijing, China). All the stained samples were observed under the microscope (DM1000, Leica Microsystems, Wetzlar, Germany).

### Gut bacteria analysis by 16S rRNA gene-based amplicon sequencing

#### Fecal DNA extraction and PCR amplification

The total bacterial DNA was isolated from fecal samples using the cetyltrimethylammonium bromide (CTAB) method. The specific experimental protocol was as follows: DNA concentration in all samples was detected by1% agarose gel. DNA was diluted to 1 ng/μL using sterile water. Extracted DNA from each sample was then used as a template to amplify the *V3-V4* region of 16S rRNA genes using PCR (21). All PCRs were performed as 30 μL reactions containing 15 μL of Phusion^®^ High-Fidelity PCR Master Mix with GC Buffer (New England Biolabs), 0.2 μM of forward and reverse primers each, and 10 ng of template DNA. The purity of PCR products was examined using electrophoresis (2% agarose gel).

#### 16S rRNA gene-based amplicon sequencing and bioinformatics analysis

The sequencing libraries were generated using TruSeq^®^ DNA PCR-Free Sample Preparation Kit (Illumina, USA) according to the manufacturer’s recommendations. Index codes were also added. The library quality was assessed on the Qubit @ 2.0 Fluorometer (Thermo Scientific). The library was then sequenced on an Illumina NovaSeq platform.

Analytical methods were used to identify bacteria and their abundances in the samples and among groups. The alpha diversity index of samples was evaluated using Mothur (Version 1.30) (22). Other related indexes, such as observed operational taxonomic units (OTUs), Chao1 richness estimator, Shannon diversity index, and Faith’s phylogenetic distance, were calculated to estimate microbial diversity within individual samples. The observed OTUs and Chao1 indexes reflect the abundance of species, while the Shannon and Simpson indexes represent the abundance and diversity of species constituting the samples. The QIIME analysis was used to analyze beta diversity, and the similarity in species diversity across the different groups of samples was compared. The beta diversities were visualized by PCoA (principal coordinate analysis) and NMDS (Non-metric multidimensional scaling, NMDS) frameworks. PCoA with Bray-Curtis distance was used to examine the separation of species and metabolites in the different samples (23). Differences in the relative abundance of the microbial features were determined by linear discriminant analysis (LDA) effect size (LEfSe) (24).

#### Spearman correlation analysis

The Spearman correlation coefficient^1^ was calculated to determine the potential correlations between the intestinal flora and different biochemical indicators. The correlations determined between the variables were displayed using heat maps. Finally, a network revealing the gut microbiota, associated markers, and disease relationships was drawn to evaluate the beneficial effects of PSCP-Fe against IDA.

#### Statistical analysis

All results were analyzed using the Statistical Product and Service Solutions (SPSS) 23.0 software (SPSS Inc., Chicago, IL, USA), and were presented as Mean ± standard deviation (SD). One-way ANOVA and Student’s *t*-test were used to assess statistical significance for multiple comparisons among the different groups. *P* < 0.05 and *p* < 0.01 were defined as statistically significant. Data visualization of results was performed using GraphPad Prism 8.0.2 software (San Diego, CA, USA).

## Results

### Structural characterization of PSCP-Fe

**FIGURE 1:**
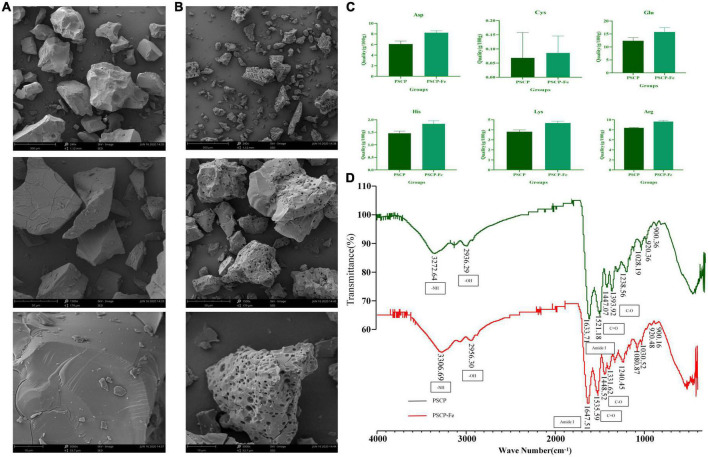
The scanning electron microscopy of PSCP **(A)** and PSCP-Fe **(B)**. Differences in amino acid content between PSCP and PSCP-Fe **(C)**. The FTIR spectroscopy of PSCP and PSCP-Fe **(D)**.

As shown in Figures 1A, B, the microstructure of PSCP-Fe was significantly different from that of PSCP. The PSCP surface was relatively flat, smooth, and voluminous. A few cracks were seen, which could have been carved by drying PSCP in the electron microscope. Conversely, the surface of PSCP-Fe was irregular and rough. The significant difference in the surface structures of PSCP and PSCP-Fe suggested the success of the chelation reaction. Amino acid composition analysis showed that PSCP and PSCP-Fe contained 17 amino acids, illustrating the integrity of amino acids during the chelation reaction (Supplementary Table 1). The contents of some amino acids, including aspartic acid (Asp), arginine (Arg), histidine (His), glutamic acid (Glu), cystine (Cys), and lysine (Lys), were increased in PSCP-Fe as compared to PSCP (Figure 1C). It has been reported that the composition and sequence of amino acids in peptides determine their ferrous-binding capacities (25). Thus, our results suggested that peptides containing the above amino acids may exhibit higher ferrous-chelating capacities (25–30).

FTIR spectroscopy was applied to reveal the chelation sites on PSCP for Fe^2+^. PSCP could generate several infrared-active vibrational modes: −NH (−3,300 cm^–1^), −OH (−3,100 cm^–1^), C-O (−1,400 cm^–1^), and C = O (−1,500 cm^–1^) (31). As shown in Figure 1D, the FTIR spectrum of PSCP-Fe presented interesting features as compared to the spectrum of PSCP. Firstly, the C-O and C = O bands at 1393.92 and 1521.18 cm^–1^ in the PSCP spectrum were shifted to 1331.62 and 1535.59 cm^–1^ after binding with Fe^2+^, which might be caused by the combination of −COO and Fe^2+^. The amide (−NH) band at 3272.64 cm^–1^ in PCSP was shifted to 3306.69 cm^–1^ in PSCP-Fe, indicating that −NH was also involved in the chelation reaction. The Amide I peak was observed at 1,600–1,700 cm^–1^, which was the absorption band of C = O stretching. In this study, the band of amide I was shifted from 1633.71 to 1647.51 cm^–1^ after Fe^2+^ chelation. Moreover, the broad absorption bands at 900, 922, and 1,020 cm^–1^, which were generated by the benzene ring of tyrosine, remained consistent in PSCP and PSCP-Fe, excluding the possibility of tyrosine being coordinated to Fe^2+^. As the above results, Asp, Arg, His, Glu, Cys, and Lys in PSCP should be more easily chelated with Fe^2+^ through functional groups of −NH and −COO.

### PSCP-Fe attenuates Low-Fe^2+^ diet-induced dysplasia and promotes iron absorption in IDA rats

**FIGURE 2:**
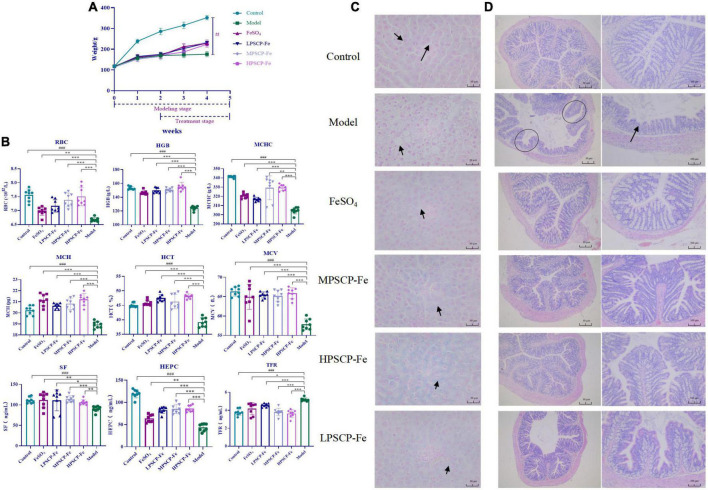
Effects of PSCP-Fe on body weight **(A)** and blood routine parameters **(B)** in IDA rats. The data in the columns are expressed as the mean ± SD (*n* = 8). Different marks (^#^, *) indicate differences at the *p* < 0.05 level. ^###^*p* < 0.001: Control vs. Model; *p < 0.05, ^**^p < 0.01, and ^***^p < 0.001: Treatments vs. Model. Liver iron staining evaluation by Prussian blue staining method **(C)**, × 400 magnification. Evaluate the histopathology changes of the rats colon tissues by H&E staining **(D)**.

Iron deficiency usually affects the growth and development of the body. In this study, rats that were given a low-Fe^2+^ diet indeed presented many undesirable morphological features as compared to the control rats. For instance, the BWs of IDA rats were significantly decreased (*p* < 0.05), this was accompanied by negative symptoms, such as dullness, unresponsiveness, and roughness of skin. These results suggested that the low-Fe^2+^ diet adversely affected the growth of rats. After 21 days of iron supplementation, the BWs in all PSCP-Fe treatment groups were significantly increased (*p* < 0.05), while the untreated IDA model and the FeSO_4_ treatment group, showed no significant difference (*p* > 0.05). In addition, there were no significant differences in BW among the three PSCP-Fe treatments (low, medium, and high), indicating that PSCP-Fe had a positive effect on the growth of IDA rats (Figure 2A).

The serum indicators in the rat groups are shown in Figure 2B. The levels of RBC and HGB, MCHC, MCH, HCT, and MCV were significantly lower in the model group compared to the control group (*p* < 0.001). After iron supplementation, these serum indicators were significantly raised (*p* < 0.05). The RBC and HGB, MCHC, HCT, and MCV were significantly increased by 1.058, 1.026, 1.028, 1.010, and 1.004% in the MPSCP-Fe group compared to the FeSO_4_ group (*p* < 0.05). In addition, RBC, HGB, MCHC, MCH, HCT, and MCV were also increased by 1.075, 1.061, 1.028, 1.002, 1.050, and 1.015% in the HPSCP-Fe group compared to the FeSO_4_ group (*p* < 0.05). Therefore, PSCP-Fe can effectively improve anemia symptoms.

Simultaneously, the SF, HEPC, and TFR levels were also determined. The contents of SF and HEPC in the Model group were significantly decreased, while TFR was up-regulated compared to the control group (*p* < 0.001). However, all iron supplement significantly reversed the above situations (*p* < 0.05). Additionally, SF and HEPC levels were increased significantly (by 1.021 and 1.335%), while TFR levels were decreased (by 1.098%) in the MPSCP-Fe group compared with FeSO_4_ group. These results showed the effective effect of PSCP-Fe resist IDA.

### PSCP-Fe can alleviate Fe^2+^ loss in the liver and colon damage

The iron deposition and pathological changes in liver tissues in each group of rats were assessed *via* Prussian blue staining assay. The rats in the IDA model group exhibited a series of adverse symptoms, including hepatocyte apoptosis and necrosis, nucleus pyknosis, hepatocyte edema, and ballooning degeneration. These pathological injuries were clearly alleviated upon supplementation with Fe^2+^, especially in MPSCP-Fe and HPSCP-Fe groups. Simultaneously, Fe^2+^ was scarcely observed in IDA rats, indicating a low-Fe^2+^ diet restricted the accumulation of Fe^2+^ in the liver. After treatment, all Fe^2+^ supplements reversed the above problem. PSCP-Fe revealed better enrichment effects of iron than FeSO_4_ at all doses. In addition, the reversal effect of HPSCP-Fe was better than that of LPSCP-Fe and MPSCP-Fe (Figure 2C), suggesting that PSCP-Fe could effectively promote the transportation and enrichment of Fe^2+^ to some extent.

The colon is critical for the transportation of Fe^2+^ from the intestine into the circulatory system (32). Fe^2+^ deficient diet caused colon damage, accelerating the process of IDA. In this study, IDA rats induced by a low-Fe^2+^ diet developed diarrhea, suggesting the occurrence of colon injury (33). HE staining was used to analyze the histology of the colon. Colon damage in the supplementary ferrous groups was much less than that observed in the IDA model group. Furthermore, colon morphology in the PSCP-Fe groups was the same as that observed in the control group, whereas colon morphology in the FeSO_4_ group was not the same as that observed in the control group (Figure 2D). These observations suggested that PSCP-Fe administration was beneficial to the colon or caused less irritation to the intestinal tract.

### The PSCP-Fe treatment restored dysbiosis in the gut microbiota in IDA rats

In this study, 24 fecal samples from the Control, Model, HPSCP-Fe, and LPSCP-Fe groups were used to evaluate the ameliorative effect of PSCP-Fe against IDA. The composition and diversity of the bacterial communities were assessed through 16S rRNA gene sequencing. After quality filtering and trimming, 1,355,946 high-quality sequences were obtained with an average of 56,497 reads per sample. The Pan/Core and rarefaction curves showed the presence of clear asymptotes, confirming the completeness of the sampling (Supplementary Figure 1).

**FIGURE 3:**
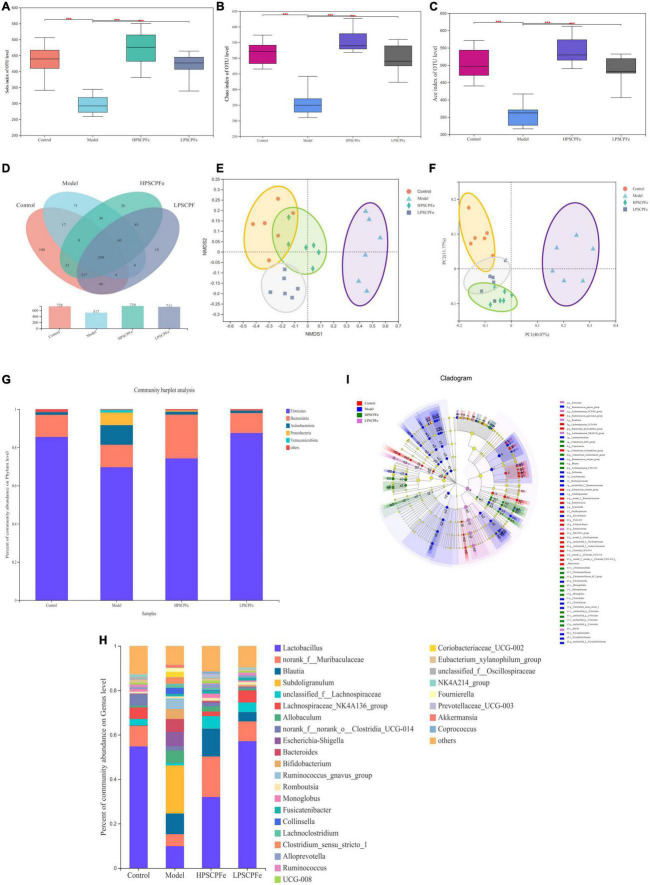
PSCP-Fe treatment restored the distribution of gut flora composition in IDA rat. **(A)** The Sobs index of OTU level. **(B)** The Chao index of OTU level. **(C)** The Ace index of OTU level. In each group, *n* = 6. ^***^p < 0.001: control or treatment vs. model group. **(D)** Venn diagram of 16S rRNA based profiling. **(E)** Non-metric multidimensional scaling (NMDS) based on the Bray-Curtis dissimilarity. **(F)** Bray-Curtis PCoA of 16S rRNA based profiling. **(G)** Relative abundance of bacterial phylum. **(H)** Relative abundance of bacterial genus. **(I)** Taxonomic cladogram generated by LEfSe analysis showing taxa significantly enriched in control (red), model (blue), HPSCP-Fe (green), LPSCP-Fe (purple), respectively. Each ring represents a taxonomic level from phylum to genus. The diameter of each dot on the ring represents the relative abundance of the taxon.

Illumina MiSeq 16S rRNA amplicon sequencing was conducted to determine the effects of PSCP-Fe treatment on gut microbiota. As shown in Figures 3A–C, the Sobs Chao index and Ace revealed that the alpha diversity in the model rats was significantly lower than that in the other groups (*p* < 0.05). However, PSCP-Fe recovered the alpha diversity relative to the IDA rats. The Venn plots showed the structural similarity and overlap of species in different groups. 728 OTUs were identified in the Control group, while only 517 OTUs were in Model group, indicating that the low-Fe^2+^ diet directly affected the composition of the intestinal flora (Figure 3D). With PSCP-Fe treatment, the numbers of OTUs were increased to 739 and 711 in HPSCP-Fe and LPSCP-Fe, respectively. Overall, the model, control, LPSCP-Fe, and HPSCP-Fe groups shared 299 OTUs with the following percentages: 41.07, 57.83, 40.46, and 42.05, indicating that the gut microbiota of the IDA model group changed significantly compare with the control group, while PSCP-Fe administration ameliorated this issue.

The beta diversity was described by PCoA analysis and NMDS, it showed that there were different clusters among the four groups, with significant differences among the clusters (Figures 3E, F). The low-Fe^2+^ diet led to a decrease in the number of OTUs in IDA rats, but the situation could be reversed with PSCP-Fe intervention. Taken together, the results suggested that PSCP-Fe treatment could modulate the IDA-induced dysbiosis of intestinal flora and restored a microbial community similar to that in the control.

### Comparison of gut microbiome at phylum and genus levels

**FIGURE 4:**
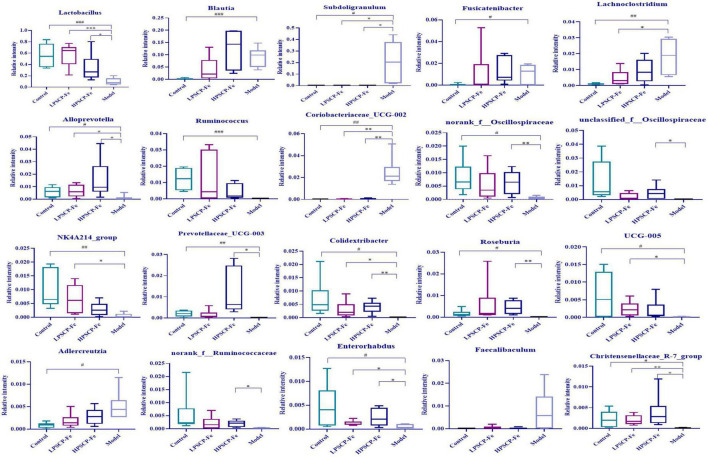
Average relative abundance of difference features flora in feces of all groups. Data are presented as mean ± SD, *n* = 6. ^#^*p* < 0.05, ^##^*p* < 0.01, ^###^*p* < 0.001: control vs. model; *p < 0.05, ^**^p < 0.01, ^***^p < 0.001: treatment vs. model.

At the phylum level (Figure 3G), *Firmicutes* and *Bacteroidota* were the two most dominant phylum with a relative abundance of 97.1% in the control group, 81.4% in the model group, 97.1% in the HPSCP-Fe group, and 98.4% in the LPSCP-Fe group. The differently abundant taxa among the experimental samples were identified using LEfSe performed at the taxonomic levels from phylum to genus, the above results is shown at Figure 3I. In addition, the low-Fe^2+^ diet could decrease the relative abundances of *Firmicutes* and *Bacteroidota*, yet PSCP-Fe fixed this deficiency. At the same time, microbiota composition at the genus levels were also identified (Figure 3H). For genus analysis, the composition of gut microbiota (greater than 0.1% abundance) is presented and summarized in Figure 4. 20 gut bacteria, such as *Lactobacillus*, *norank_f_Muribaculaceae*, *Blautia*, *Subdoligranulum*, *unclassified_f Lachnospiraceae*, *NK4A214_group*, *Fournierella*, *Prevotellaceae_UCG-003*, *Akkermansia*, and *Coprococcus*, were significantly altered (*p* < 0.05). The Kruskal Wallis test for the significance of difference was performed on genus-level OTUs and genera (*p* < 0.05). The 13 bacteria in IDA rats were significantly reduced as compared to the control (*p* < 0.05), and 7 bacteria had increased (*p* < 0.05). *Lactobacillus, Alloprevotella, unclassified_of_Oscillospiraceae, NK4A214_group, Prevotellaceae_UCG-003, Colidextribacter, Coriobacteriaceae_UCG-005, Enterorhabdus*, and *Christensenellaceae_R-7_group* were reduced in IDA rats upon PSCP-Fe treatment. However, two simultaneous doses of PSCP-Fe significantly increased the relative abundances of 5 out of 9 bacteria (*p* < 0.05). In addition, LPSCP-Fe and HPSCP-Fe could downgrade the relative abundances of *Subdoligranulum* and *Coriobacteriaceae_UCG-002* (*p* < 0.05). *Lactobacillus, Alloprevotella, unclassified_of_Oscillospiraceae, NK4A214_group, Prevotellaceae_UCG-003, Colidextribacter, Coriobacteriaceae_UCG-005, Enterorhabdus*, and *Christensenellaceae_R-7_group* might benefit the action of PSCP-Fe in the treatment of IDA. However, *Subdoligranulum* and *Coriobacteriaceae_UCG-002* contribute to the occurrence of IDA.

### The correlation between hematological indicators and intestinal microbiota

**FIGURE 5:**
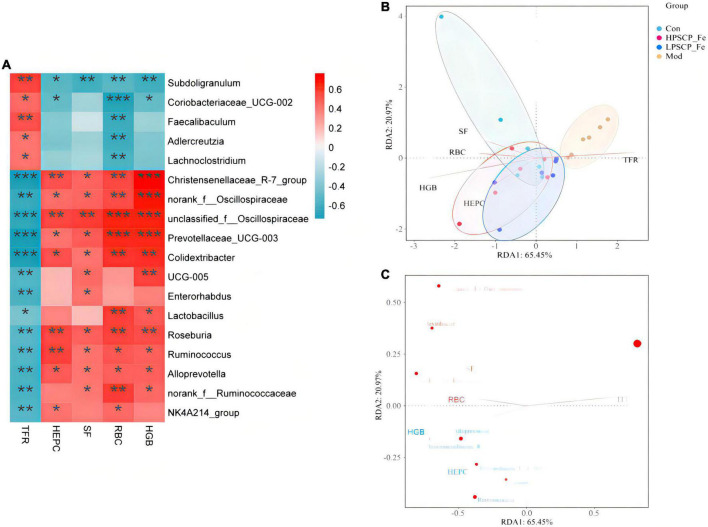
Correlation analysis between related factors and microbiota at the genus level **(A)**. Spearman’s correlation analyses were used to analyze the relative abundance (%) between the specific genus in each treatment group. 0.01 < *p ≤ 0.05, 0.001 < **p ≤ 0.01, ***p ≤ 0.001. **(B)** The db-RDA analysis result in different groups. **(C)** The db-RDA analysis result of gut flora and the related factors.

To determine the regulatory mechanisms of different microbiota, the Spearman’s correlation analysis was used. The relative abundances of specific microflora were correlated with hematology-related indicators as shown in Figure 5A. Eighteen bacterial genus showed correlations with cytokines at different levels, among which five bacteria correlated positively with TFR (*p* < 0.05). Based on the correlation coefficients, correlations with TFR of different species were in the order of *Subdoligranulum* (*p*≈ 0.003) > *Faecalibaculum* (*p*≈ 0.009) > *Coriobacteriaceae_UCG-002* (*p*≈ 0.027) > *Lachnoclostridium* (*p*≈ 0.046) > *Adlercreutzia* (*p*≈ 0.047). In addition, these gut bacteria presented negative correlations with RBC, HGB, HEPC, and SF. However, only *Subdoligranulum* was significantly negatively correlated with all four factors (*p* < 0.05), where the correlation coefficients were 0.009 (RBC), 0.002 (HGB), 0.01 (HEPC), and 0.002 (SF), showing that *Subdoligranulum* strongly promoted the occurrence of IDA. In addition, *Faecalibaculum*, *Coriobacteriaceae_UCG-002, Adlercreutzia*, and *Lachnoclostridium* showed significantly negative correlations with RBC. These five gut bacteria might be regarded as inhibitors of RBC recovery in IDA rats.

For the next analysis, 13 gut bacteria showed negative correlations with TFR and positive correlations with RBC, HGB, SF, and HEPC. Among these, *Christensenellaceae*_R-7_group, unclassified_f_*Oscillospiraceae*, *Prevotellaceae*_UCG-003, and *Colidextribacter* presented significantly negative correlations (0.0001 < *p* < 0.001) compared to the other 9 bacteria, indicating that these microbes seemed to be closely related to the inhibition of TFR expression. Nevertheless, 8 of the 13 bacteria, including *Christensenellaceae*_R-7_group, norank_f_*Oscillospiraceae*, unclassified_f_*Oscillospiraceae*, *Prevotellaceae*_UCG-003, *Colidextribacter*, *Roseburia*, *Ruminococcus*, and *Alloprevotella*, showed significantly positive correlations with RBC, HGB, HEPC, and SF (*p* < 0.05). The above bacteria might offer incredible functions for the recovery of active factors. Based on these correlation data, we found that different bacteria might play different roles in regulating cytokines. For cytokines, the correlations between HGB and *Christensenellaceae*_R-7_group, norank_f_*Oscillospiraceae*, unclassified_f_*Oscillospiraceae*, and *Prevotellaceae*_UCG-003 (0.0001 < *p* < 0.001) were 10 times stronger than the correlations with other bacteria (0.001 < *p* < 0.01). Moreover, norank_f_*Oscillospiraceae* and unclassified_f_*Oscillospiraceae* (0.0001 < *p* < 0.001) were more focused on restoring the RBC levels than the other 5 bacterial phyla (0.001 < *p* < 0.05). However, for SF, only unclassified_f_*Oscillospiraceae* showed a more intensive correlation (*p* < 0.01) than the other bacteria. Next, *Christensenellaceae*_R-7_group, unclassified_f_*Oscillospiraceae*, *Roseburia*, and *Ruminococcus* were found to have obviously correlations with HEPC (0.001 < *p* < 0.01).

Putting aside the above phenomenon, we found that the same bacteria revealed differential correlations with different cytokines. The negative correlation parameters of *Subdoligranulum* and SF, RBC, and HGB (0.001 < *p* < 0.01) were significantly higher than the correlation with HEPC (0.01 < *p* < 0.05). Thus, this bacterium was able to inhibit the expression of SF, RBC, and HGB, while inhibiting the expression of HEPC. In addition, *Christensenellaceae*_R-7_group could reverse HGB levels more (0.0001 < *p* < 0.001) than the levels of SF, RBC, and HEPC (0.001 < *p* < 0.05). Norank_f_*Oscillospiraceae* also exhibited the same property (0.0001 < *p* < 0.001). Both unclassified_f_*Oscillospiraceae* and *Prevotellaceae*_UCG-003, restored the levels of HGB and RBC (0.0001 < *p* < 0.001), rather than the levels of SF and HEPC (*p* < 0.01). The correlation gradation of *Roseburia* and cytokines was RBC (*p*≈ 0.004) > HEPC (*p*≈ 0.005) > HGB (*p*≈ 0.007) > SF (*p*≈ 0.02), illustrating that these genera might be of importance in increasing the levels of RBC, HEPC, and HGB. However, *Ruminococcus* seemed to be more associated with the regulation of HEPC (0.001 < *p* < 0.01). *Alloprevotella* did not offer significant correlations with any of the four factors.

In addition to the above-mentioned microbiota, some probiotics were found to be associated with sporadic cytokines. *Lactobacillus* showed a positive correlation between RBC and HGB (0.001 < *p* < 0.05), and its ability to regulate RBC might be more prominent than HGB. *Coriobacteriaceae*_UCG-005, a Gram-negative bacterium, was significantly positively correlated with HGB and SF (*p* < 0.05). Coincidentally, two bacteria were negatively correlated with TFR (*p* < 0.05). These results suggested that *Coriobacteriaceae*_UCG-005 and *Lactobacillus* should alleviate IDA by inhibiting the expression of TFR and increasing the levels of HGB, RBC, and SF.

The distance-based redundancy analysis (db-RDA) was performed to determine whether the cytokines correlated with microbiota (Figures 5B, C). The levels of SF, HGB, RBC, and HEPC were positively associated with each other, and negatively correlated with TFR. In addition, *Subdoligranulum* and *Christensenellaceae*_R-7_group (*p* < 0.05) were closely associated with cytokines (*p* < 0.05) than the other microbiota. Thus, the *Subdoligranulum* and *Christensenellaceae*_R-7_group should be potential microbial markers for PSCP-Fe action in the treatment of IDA.

## Discussion

The active peptides from natural sources, such as wheat, egg white, and soy, often carry trace elements with incredible functions (4, 34). In this study, we chose pig skin as the chelating agent of Fe^2+^ to obtain PSCP-Fe. A range of analytical methods was then used to determine the chelating property of PSCP and Fe^2+^. It was found that the surface structure of PSCP-Fe was significantly different from that of PSCP. Pore traces of PCSP-Fe were likely to be left by the chelation reaction of PSCP with Fe^2+^, which has been reported in previous literature (35). Peptides containing Asp, Arg, His, Glu, Cys, and Lys residues exhibit higher iron-chelating capacity. Among these, Asp and Glu are acidic amino acids that can contribute more −COO to achieve chelation with Fe^2+^ compared to neutral amino acids. These chelating abilities have been confirmed in Fe^2+^-binding peptides in whey protein hydrolysates and soy protein (34, 36). Similarly, Arg, His, and Lys are basic amino acids that can provide −NH groups to combine with Fe^2+^ (37). These results are similar to the observation of Greve et al. (27), Johnson et al. (29), and De la Hoz et al. (30). Interestingly, Cys has been recognized as an important amino acid that has excellent ferrous binding capacity because of its functional groups (−NH, −COO, and −SH), especially when it is derived from digested meat protein (38, 39). FTIR analysis confirmed that the functional groups of these amino acids were involved in chelation with Fe^2+^. The absorption bands of −NH and −COO were changed significantly, showing the authenticity of the functional groups involved in the chelation reaction.

An early meta-analysis revealed that 42.7% of women in developing countries experience anemia during pregnancy, which is associated with a significantly higher risk of low birth weight in infants (40). Underweight and malnourished infants and toddlers are more susceptible to IDA. In this experiment, 4-week-old SD rats (weighing 110 g) were equivalent to a 4–6-years-old human child,^2^ and thus, were used to evaluate the effect of PSCP-Fe against IDA. The levels of RBC, HGB, HCT, MCV, and MCH in the blood can provide diagnostic evidence of IDA. We found that the low-Fe^2+^ diet could indeed cause significant changes in the above parameters (*p* < 0.05), while PSCP-Fe could reverse their deficiencies (*p* < 0.05). These results indicated that PSCP-Fe may treat IDA by interfering with the production of HGB and RBCs.

In addition, the levels of the secreted factors, including HEPC, TFR, and SF, were determined. HEPC is an antimicrobial peptide found in blood and urine. It is predominantly expressed in the liver and is regulated based on the hepatic iron stores (41). TFR is a liver-derived protein that can bind up to two iron atoms. Iron-laden transferrin delivers the metal to most cells upon binding to TFR (42). TFR mediates cellular iron uptake through clathrin-dependent endocytosis of iron-loaded transferrin, thus playing a key role in iron homeostasis. Moreover, SF is synthesized by the liver and can transport iron to all tissues through an affinity reaction, while transporting excess iron to the liver for storage. SF level is also widely measured as an indicator of iron status (43). The present study showed that PSCP-Fe could significantly increase the levels of HEPC and SF, while downgrading the levels of TFR (*p* < 0.05) compared to untreated IDA rats; its effect was thus better than FeSO_4_. These results were similar to the observations of Mantadakis et al. (3) and Xiao et al. (44). Certainly, the colonic damage caused by additive should not be ignored. The free Fe^2+^ can cause side effects, such as colon and liver damage and inflammation. Compared with the free Fe^2+^, the chelate of peptide and Fe^2+^ shows miraculous stability in a series of biomimetic experiments, and this chelate can only be degraded reluctantly in the duodenum. This suggests that the destruction of colon and intestine by chelate of Fe^2+^ and peptide is negligible (45, 46). In this research, we found that PSCP-Fe was beneficial to the colon or caused less irritation to the intestinal tract, and the reversal was much better than FeSO_4_, which was recommended by pharmacopeias and food additive regulations in many countries. These results indicated the security of PSCP-Fe. Surprisingly, in histopathology experiments, more Fe^2+^ was noted to be transported to the liver after supplementation by PSCP-Fe, confirming the therapeutic effect on IDA. Two possible mechanisms for the enhancement effect of peptides in the non-heme Fe^2+^ bioavailability have been reported, on the one hand, the peptide can intercept the incorporation between Fe^2+^ and non-nutritional factors in diet, such as phatic acid, oxalate or tannin. On the other hand, peptide can increase Fe^2+^ absorption by maintaining the morphology of Fe^2+^ and enhancing chelating capacity with Fe^2+^ (47). However, the role of PSCP in Fe^2+^ absorption was ambiguous in this study. In addition, whether the gut microbiota is related to the absorption of PSCP-Fe was not discussed in this study. These problems will be deeply dissected in our next work.

**FIGURE 6:**
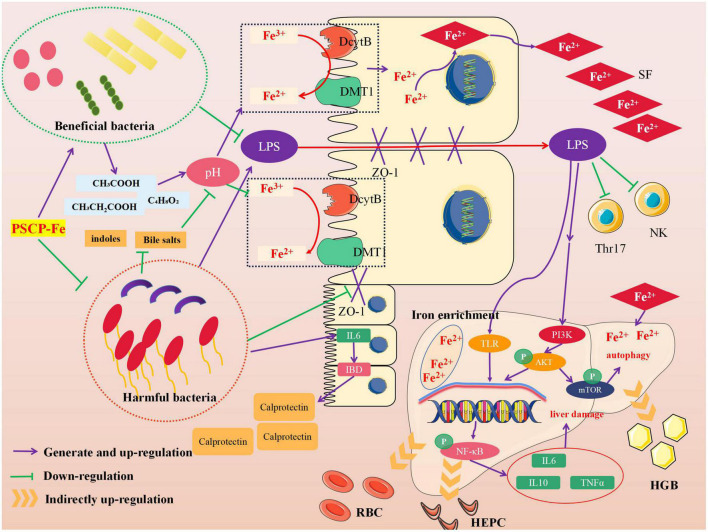
Intervention mechanism of PSCP-Fe on IDA rats.

Neurological analysis suggested that the key role of iron absorption should be the intestinal solubility of oral iron (48). Gut microbiota is critical for maintaining iron homeostasis in the intestine. *Escherichia*, *Salmonella*, *Faecalibaculum*, and *Vibrio cholerae* have reported the ability to lock iron by generating siderophores, therefore, iron from daily diets cannot be transported into the circulatory system, leading to IDA (49). In addition, the imbalance in iron content caused by intestinal flora may increase the levels of fecal calprotectin, which is released by neutrophils and phagocytes, causing inflammation of the intestine (50). Specific bacterial genera that mediate the development of intestinal inflammation, such as *Adlercreutzia*, *Coriobacteriaceae*, *Faecalibaculum*, and *Clostridium*, can release many harmful metabolites (e.g., secondary bile salts, hydrogen sulfide, and indoles), accelerating inflammation and cancer-induction in intestinal tissue (51). These pathological conditions may also reduce the expression of the duodenal cytochrome B (DcytB) and divalent metal transporter 1 (DMT1) by disrupting the intestinal mechanical barrier, and their coupled action is required for Fe^2+^ absorption. It must be noted that the above bacteria are able to restrain the conversion of Fe^3+^ to Fe^2+^ by mediating intestinal pH, indirectly causing Fe^2+^ deficiency *in vivo*. In addition, the zonula occludens-1 (ZO-1) and occludin, which reflect the integrity of the intestinal mechanical barrier, can also be destroyed due to long-term erosion by harmful bacteria and their metabolites. In this case, lipopolysaccharide (LPS) entering the body can lead to tissue inflammation by increasing inflammatory factors, such as interleukin 6 (IL-6), interleukin 10 (IL-10), and tumor necrosis factor α (TNF-α). The latter activates the TLR/NF-κB pathway and inhibits the differentiation of immune cells, such as Thr17 and NK (52). Figure 6 shows the intervention mechanism of PSCP-Fe on IDA rats. In this study, we found that a low-Fe^2+^ diet altered the structure of gut microbiota and increased the relative abundances of *Lachnoclostridium*, *Adlercreutzia*, *Faecalibaculum, Coriobacteriaceae*_UCG-002, and *Subdoligranulum*, which is consistent with the literature (53). Some beneficial bacteria were also recovered by administering PSCP-Fe. Probiotics can alleviate IDA by making use of compound pathways that promote Fe^2+^ absorption. For instance, bacteria that produce (SCFAs) and lactic acid derivatives can restore the pH of the intestinal lumen, thereby promoting the conversion of Fe^3+^ to Fe^2+^, and they can also repair intestinal tissue and up-regulate the expression of related receptors to increase Fe^2+^ absorption. Surprisingly, SCFAs can inhibit the spread of LPS, and thus, eliminate inflammation. Here, PSCP-Fe increased the relative abundances of *Blautia*, *Alloprevotella*, *Ruminococcus*, *Oscillospiraceae*, *Prevotellaceae*_UCG-003, *Roseburia*, norank_f_*Ruminococcaceae*, and *Christensenellaceae*_R-7_group, which are producers of SCFAs, including butyric acid, propionic acid, and acetic acid. A previous report has indicated that *Lactobacillus*, which is the main probiotic found in human microbiota, exhibits a remarkable Fe^3+^-reducing activity due to its metabolite, P-hydroxyphenyllactic acid, and increases Fe^2+^ absorption through DMT1. Coincidentally, PSCP-Fe increased the relative abundance of *Lactobacillus* in IDA rats. Liver histopathology should reveal liver damage caused by harmful bacteria and their metabolites, along with injury to intestinal tissue. After PSCP-Fe treatment, the relative abundances of beneficial bacteria were positively correlated with iron enrichment and tissue repair. The fortification of iron also contributes to the reduction in beneficial bacteria and is directly associated with intestinal inflammation. However, beneficial bacteria did not decrease upon intervention with PSCP-Fe, demonstrating the safety of PSCP-Fe and the potential activities of PSCP.

Red blood cells, HGB, and SF are important indicators of iron in the body. HEPC and TFR are critical parameters for iron homeostasis and immune response *in vivo*. The Spearman correlation analysis was utilized to determine whether the five factors were associated with differential microflora. Five gut bacteria were positively correlated with TFR, and 13 gut bacteria showed positive correlations in RBC, HGB, SF, and HEPC. *Subdoligranulum and Christensenellaceae*_R-7_group were more closely related to the above five factors. *Subdoligranulum* is a butyric acid-producing bacteria, which plays an important role in alleviating obesity and Type-2 diabetes (54). However, we found that this bacterium did not increase iron content in the body by producing butyric acid; instead, it promoted iron release based on the positive correlation with TFR, highlighting its indistinct function in *Subdoligranulum*. Conversely, *Christensenellaceae*_R-7_group, which is known as special probiotics, has also been related to a healthy status in the context of different diseases, including obesity and inflammation. In a study on intestinal inflammation, *Christensenellaceae* _R-7_group remained depleted in individuals with Crohn’s disease (CD) and ulcerative colitis (UC), which are two major subtypes of inflammatory bowel disease (IBD) (55). Therefore, *Christensenellaceae* _R-7_group may increase Fe^2+^ absorption by repairing intestinal mucosa and upregulating receptor expression. These changes would in turn alleviate the low levels of HGB, RBC, and SF caused by IDA. This group can also up-regulate the expression of certain factors and increase HEPC levels. However, the mechanisms behind these changes have not yet been revealed, and the correlation between *Christensenellaceae* _R-7_group, PSCP and Fe^2+^ has been also hidden. Therefore, the relevant problems should be studied in follow-up experiments.

## Data availability statement

The original contributions presented in this study are publicly available. This data can be found here: https://www.ncbi.nlm.nih.gov/bioproject/PRJNA796635.

## Ethics statement

The animal study was reviewed and approved by the Institutional Animal Care and Use Committee at Bin Zhou Medical University (approval certificate number: 2020-090).

## Author contributions

SW, LD, and JZ developed the idea and designed the research. SJ and WD performed the experiments and wrote the draft of the manuscript. SW, ZZ, JX, and HL contributed to the revise the writing. All authors read and approved the submitted version.

## Footnotes

1
https://www.bioincloud.tech/

2
https://www.taletn.com/rats/age/

## References

[1] Stelle I Kalea A Pereira D. Iron deficiency anaemia: experiences and challenges. *Proc Nutr Soc.* (2019) 78:19–26. 10.1017/S0029665118000460 29986781

[2] Baumgartner J Barth-Jaeggi T. Iron interventions in children from low-income and middle-income populations: benefits and risks. *Curr Opin Clin Nutr Metab Care.* (2015) 18:289–94. 10.1097/MCO.0000000000000168 25807351

[3] Mantadakis E Chatzimichael E Zikidou P. Iron deficiency anemia in children residing in high and low-income countries: risk factors, prevention, diagnosis and therapy. *Mediterr J Hematol Infect Dis.* (2020) 12:e2020041. 10.4084/MJHID.2020.041 32670519 PMC7340216

[4] Li B He H Shi W Hou T. Effect of duck egg white peptide-ferrous chelate on iron bioavailability in vivo and structure characterization. *J Sci Food Agric.* (2019) 99:1834–41. 10.1002/jsfa.9377 30255570

[5] Zarei M Ghanbari R Tajabadi N Abdul-Hamid A Bakar F Saari N. Generation, fractionation, and characterization of iron-chelating protein hydrolysate from palm kernel cake proteins. *J Food Sci.* (2016) 81:C341–7. 10.1111/1750-3841.13200 26720491

[6] Lin H Deng S Huang S Li Y Song R. The effect of ferrous-chelating hairtail peptides on iron deficiency and intestinal flora in rats. *J Sci Food Agric.* (2016) 96:2839–44. 10.1002/jsfa.7452 26425939

[7] Torres-Fuentes C Alaiz M Vioque J. Iron-chelating activity of chickpea protein hydrolysate peptides. *Food Chem.* (2012) 134:1585–8. 10.1016/j.foodchem.2012.03.112 25005984

[8] Das N Schwartz A Barthel G Inohara N Liu Q Sankar A Microbial metabolite signaling is required for systemic iron homeostasis. *Cell Metab.* (2020) 31:115.e–30.e. 10.1016/j.cmet.2019.10.005 31708445 PMC6949377

[9] Qi X Zhang Y Guo H Hai Y Luo Y Yue T. Mechanism and intervention measures of iron side effects on the intestine. *Crit Rev Food Sci Nutr.* (2020) 60:2113–25. 10.1080/10408398.2019.1630599 31232087

[10] Yilmaz B Li H. Gut microbiota and iron: the crucial actors in health and disease. *Pharmaceuticals.* (2018) 11:98. 10.3390/ph11040098 30301142 PMC6315993

[11] Oliphant K Allen-Vercoe E. Macronutrient metabolism by the human gut microbiome: major fermentation by-products and their impact on host health. *Microbiome.* (2019) 7:91. 10.1186/s40168-019-0704-8 31196177 PMC6567490

[12] Jiang H Zhang W Chen F Zou J Chen W Huang G. Purification of an iron-binding peptide from scad (*Decapterus maruadsi*) processing by-products and its effects on iron absorption by Caco-2 cells. *J Food Biochem.* (2019) 43:e12876. 10.1111/jfbc.12876 31353718

[13] Rosenberg L Bagger C Janfelt C Haedersdal M Olesen U Lerche CMA. Comparison of human and porcine skin in laser-assisted drug delivery of chemotherapeutics. *Lasers Surg Med.* (2021) 53:162–70. 10.1002/lsm.23344 33161610

[14] Hwang J Jeong H Lee N Hur S Lee N Han J Ex vivo live full-thickness porcine skin model as a versatile in vitro testing method for skin barrier research. *Int J Mol Sci.* (2021) 22:657. 10.3390/ijms22020657 33440780 PMC7827261

[15] He L Lan W Zhao Y Chen S Liu S Cen L Characterization of biocompatible pig skin collagen and application of collagen-based films for enzyme immobilization. *RSC Adv.* (2020) 10:7170–80. 10.1039/c9ra10794k 35493877 PMC9049748

[16] Ma X Liu C Song W Che S Wang C Feng X Evaluating the efficacy of a ferrous-ion-chelating peptide from Alaska pollock frame for the improvement of iron nutritional status in rats. *Food Funct.* (2019) 10:4888–96. 10.1039/c9fo00310j 31339120

[17] Li C Cui Z Li Z Gao L Zhang C Li D Determination of mineral elements in nanyang mugwort (*Artemisia argyi*) leaves harvested from different crops by inductively coupled plasma mass spectrometry and inductively coupled plasma atomic emission spectrometry. *Chem Pharm Bull.* (2021) 69:411–3. 10.1248/cpb.c20-00875 33518581

[18] Lee ES Kwon M-H Kim HM Woo HB Ahn CM Chung CH. Curcumin analog CUR5-8 ameliorates nonalcoholic fatty liver disease in mice with high-fat diet-induced obesity. *Metabolism.* (2020) 103:154015. 10.1016/j.metabol.2019.154015 31758951

[19] Nagappan A Kim J Jung D Jung M. Cryptotanshinone from the *Salvia miltiorrhiza* bunge attenuates ethanol-induced liver injury by activation of AMPK/SIRT1 and Nrf2 signaling pathways. *Int J Mol Sci.* (2019) 21:265. 10.3390/ijms21010265 31906014 PMC6981483

[20] Jun J Choi J Bae S Oh S Kim G. Decreased C-reactive protein induces abnormal vascular structure in a rat model of liver dysfunction induced by bile duct ligation. *Clin Mol Hepatol.* (2016) 22:372–81. 10.3350/cmh.2016.0032 27729629 PMC5066379

[21] Pang J Ma S Xu X Zhang B Cai Q. Effects of rhizome of *Atractylodes* Koreana (Nakai) Kitam on intestinal flora and metabolites in rats with rheumatoid arthritis. *J Ethnopharmacol.* (2021) 281:114026. 10.1016/j.jep.2021.114026 33727111

[22] Li Q Cui Y Xu B Wang Y Lv F Li Z Main active components of Jiawei Gegen Qinlian decoction protects against ulcerative colitis under different dietary environments in a gut microbiota-dependent manner. *Pharmacol Res.* (2021) 170:105694. 10.1016/j.phrs.2021.105694 34087350

[23] Erawijantari P Mizutani S Shiroma H Shiba S Nakajima T Sakamoto T Influence of gastrectomy for gastric cancer treatment on faecal microbiome and metabolome profiles. *Gut.* (2020) 69:1404–15. 10.1136/gutjnl-2019-319188 31953253 PMC7398469

[24] Yang X Liu D Ren H Zhang X Zhang J Yang X. Effects of sepsis and its treatment measures on intestinal flora structure in critical care patients. *World J Gastroenterol.* (2021) 27:2376–93. 10.3748/wjg.v27.i19.2376 34040329 PMC8130038

[25] Sun N Cui P Li D Jin Z Zhang S Lin S. Formation of crystalline nanoparticles by iron binding to pentapeptide (Asp-His-Thr-Lys-Glu) from egg white hydrolysates. *Food Funct.* (2017) 8:3297–305. 10.1039/c7fo00843k 28832063

[26] Kim S Seo I Khan M Ki K Lee W Lee H Enzymatic hydrolysis of heated whey: iron-binding ability of peptides and antigenic protein fractions. *J Dairy Sci.* (2007) 90:4033–42. 10.3168/jds.2007-0228 17699019

[27] Greve J Pinkham A Thompson Z Cowan J. Active site characterization and activity of the human aspartyl (asparaginyl) β-hydroxylase. *Metallomics.* (2021) 13:mfab056. 10.1093/mtomcs/mfab056 34543426

[28] Efsa Panel on Additives and Products or Substances used in Animal Feed (Feedap), Bampidis V Azimonti G Bastos M Christensen H Dusemund B Efficacy of iron chelates of lysine and glutamic acid as feed additive for all animal species. *EFSA J.* (2020) 18:e06164. 10.2903/j.efsa.2020.6164 32874339 PMC7448030

[29] Johnson E Russo M Nye D Schlessman J Lecomte J. Lysine as a heme iron ligand: a property common to three truncated hemoglobins from *Chlamydomonas reinhardtii*. *Biochim Biophys Acta Gen Subj.* (2018) 1862:2660–73. 10.1016/j.bbagen.2018.08.009 30251657 PMC6214630

[30] De la Hoz L Ponezi A Milani R Nunes da Silva V Sonia de Souza A Bertoldo-Pacheco M. Iron-binding properties of sugar cane yeast peptides. *Food Chem.* (2014) 142:166–9. 10.1016/j.foodchem.2013.06.133 24001827

[31] Yang S Zhang Q Yang H Shi H Dong A Wang L Progress in infrared spectroscopy as an efficient tool for predicting protein secondary structure. *Int J Biol Macromol.* (2022) 206:175–87. 10.1016/j.ijbiomac.2022.02.104 35217087

[32] Anderson G Frazer D. Current understanding of iron homeostasis. *Am J Clin Nutr.* (2017) 106(Suppl. 6):1559S–66S. 10.3945/ajcn.117.155804 29070551 PMC5701707

[33] Jie F Xiao S Qiao Y You Y Feng Y Long Y Kuijieling decoction suppresses NLRP3-mediated pyroptosis to alleviate inflammation and experimental colitis in vivo and in vitro. *J Ethnopharmacol.* (2021) 264:113243. 10.1016/j.jep.2020.113243 32781258

[34] Zhu S Zheng Y He S Su D Nag A Zeng Q Novel zn-binding peptide isolated from soy protein hydrolysates: purification, structure, and digestion. *J Agric Food Chem.* (2021) 69:483–90. 10.1021/acs.jafc.0c05792 33370528

[35] Sun R Liu X Yu Y Miao J Leng K Gao H. Preparation process optimization, structural characterization and in vitro digestion stability analysis of Antarctic krill (*Euphausia superba*) peptides-zinc chelate. *Food Chem.* (2021) 340:128056. 10.1016/j.foodchem.2020.128056 33032152

[36] Athira S Mann B Sharma R Pothuraju R Bajaj R. Preparation and characterization of iron-chelating peptides from whey protein: an alternative approach for chemical iron fortification. *Food Res Int.* (2021) 141:110133. 10.1016/j.foodres.2021.110133 33642000

[37] Wu J Cai X Tang M Wang S. Novel calcium-chelating peptides from octopus scraps and their corresponding calcium bioavailability. *J Sci Food Agric.* (2019) 99:536–45. 10.1002/jsfa.9212 29931683

[38] Park K Sim I Ko H Lim Y. Gamma aminobutyric acid increases absorption of glycine-bound iron in mice with iron deficiency anemia. *Biol Trace Elem Res.* (2020) 197:628–38. 10.1007/s12011-020-02027-9 31927755

[39] Ye J Wang S Zhang P Nabi M Tao X Zhang H L-cysteine addition enhances microbial surface oxidation of coal inorganic sulfur: complexation of cysteine and pyrite, inhibition of jarosite formation, environmental effects. *Environ Res.* (2020) 187:109705. 10.1016/j.envres.2020.109705 32474315

[40] Rahman M Abe S Rahman M Kanda M Narita S Bilano V Maternal anemia and risk of adverse birth and health outcomes in low- and middle-income countries: systematic review and meta-analysis. *Am J Clin Nutr.* (2016) 103:495–504. 10.3945/ajcn.115.107896 26739036

[41] Böser P Mordashova Y Maasland M Trommer I Lorenz H Hafner M Quantification of hepcidin-related iron accumulation in the rat liver. *Toxicol Pathol.* (2016) 44:259–66. 10.1177/0192623315623866 26839325

[42] Gammella E Buratti P Cairo G Recalcati S. The transferrin receptor: the cellular iron gate. *Metallomics.* (2017) 9:1367–75. 10.1039/c7mt00143f 28671201

[43] Wang F Xie Z Ye X Deng S Hu Y Guo X Effectiveness of treatment of iron deficiency anemia in rats with squid ink melanin-Fe. *Food Funct.* (2014) 5:123–8. 10.1039/c3fo60383k 24292561

[44] Xiao C Lei X Wang Q Du Z Jiang L Chen S Effects of a tripeptide iron on iron-deficiency anemia in rats. *Biol Trace Elem Res.* (2016) 169:211–7. 10.1007/s12011-015-0412-6 26109335

[45] Udechukwu M Collins S Udenigwe C. Prospects of enhancing dietary zinc bioavailability with food-derived zinc-chelating peptides. *Food Funct.* (2016) 7:4137–44. 10.1039/c6fo00706f 27713952

[46] Qu W Feng Y Xiong T Li Y Wahia H Ma H. Preparation of corn ACE inhibitory peptide-ferrous chelate by dual-frequency ultrasound and its structure and stability analyses. *Ultrason Sonochem.* (2022) 83:105937. 10.1016/j.ultsonch.2022.105937 35144194 PMC8844830

[47] Li Y Jiang H Huang G. Protein hydrolysates as promoters of non-haem iron absorption. *Nutrients.* (2017) 9:609. 10.3390/nu9060609 28617327 PMC5490588

[48] Milto I Suhodolo I Prokopieva V Klimenteva T. Molecular and cellular bases of iron metabolism in humans. *Biochemistry.* (2016) 81:549–64. 10.1134/S0006297916060018 27301283

[49] Seyoum Y Baye K Humblot C. Iron homeostasis in host and gut bacteria – a complex interrelationship. *Gut Microbes.* (2021) 13:1–19. 10.1080/19490976.2021.1874855 PMC7872071 33541211

[50] Liu B Pan X Liu Z Han M Xu G Dai X Fecal microbiota as a noninvasive biomarker to predict the tissue iron accumulation in intestine epithelial cells and liver. *FASEB J.* (2020) 34:3006–20. 10.1096/fj.201901635RR 31912587

[51] Schepici G Silvestro S Bramanti P Mazzon E. The gut microbiota in multiple sclerosis: an overview of clinical trials. *Cell Transplant.* (2019) 28:1507–27. 10.1177/0963689719873890 31512505 PMC6923550

[52] Low K Smith S Abbott D Boraston A. The glycoconjugate-degrading enzymes of *Clostridium perfringens*: tailored catalysts for breaching the intestinal mucus barrier. *Glycobiology.* (2021) 31:681–90. 10.1093/glycob/cwaa050 32472136

[53] Lin H Zeng C Shui S Zhang B. Effects of Fe (II)-chelating hairtail protein hydrolysates on the immune characteristics and intestinal microorganisms in loach (*Misgurnus anguillicaudatus*). *Aquac Rep.* (2021) 19:100630. 10.1016/j.aqrep.2021.100630

[54] Chen W Zhang M Guo Y Wang Z Liu Q Yan R The profile and function of gut microbiota in diabetic nephropathy. *Diabetes Metab Syndr Obes.* (2021) 14:4283–96. 10.2147/DMSO.S320169 34703261 PMC8541750

[55] Mancabelli L Milani C Lugli G Turroni F Cocconi D van Sinderen D Identification of universal gut microbial biomarkers of common human intestinal diseases by meta-analysis. *FEMS Microbiol Ecol.* (2017) 93:fix153. 10.1093/femsec/fix153 29126267

