# Supplementary material for: A new iron supplement: The chelate of pig skin collagen peptide and Fe2+ can treat iron-deficiency anemia by modulating intestinal flora
Source: Front Nutr. 2022 Dec 22;9:1055725. doi: 10.3389/fnut.2022.1055725 (PMC9815456; doi:10.3389/fnut.2022.1055725)
Supplement: Supplementary file 1 [file Data_Sheet_1.doc]

**Supplementary Table 1** The relative contents of amino acid in PSCP and PSCP-Fe.

| Amino acid | PSCP (g/100g) | PSCP-Fe (g/100g) |
| --- | --- | --- |
| Asp | 6.0989 | 8.2276 |
| Thr | 2.0765 | 2.0649 |
| Ser | 3.0518 | 3.0833 |
| Glu | 12.3507 | 15.7778 |
| Gly | 24.3160 | 24.2595 |
| Ala | 11.7074 | 10.4249 |
| Cys | 0.0680 | 0.0856 |
| Val | 3.1690 | 2.2669 |
| Met | 0.8507 | 0.5042 |
| Ile | 1.5303 | 1.0659 |
| Leu | 3.8883 | 2.4708 |
| Tyr | 0.9847 | 0.9388 |
| Phe | 2.6059 | 1.9907 |
| His | 1.4575 | 1.8362 |
| Lys | 3.8035 | 4.6715 |
| Arg | 8.3835 | 9.6472 |
| Pro | 13.6559 | 10.6846 |

**
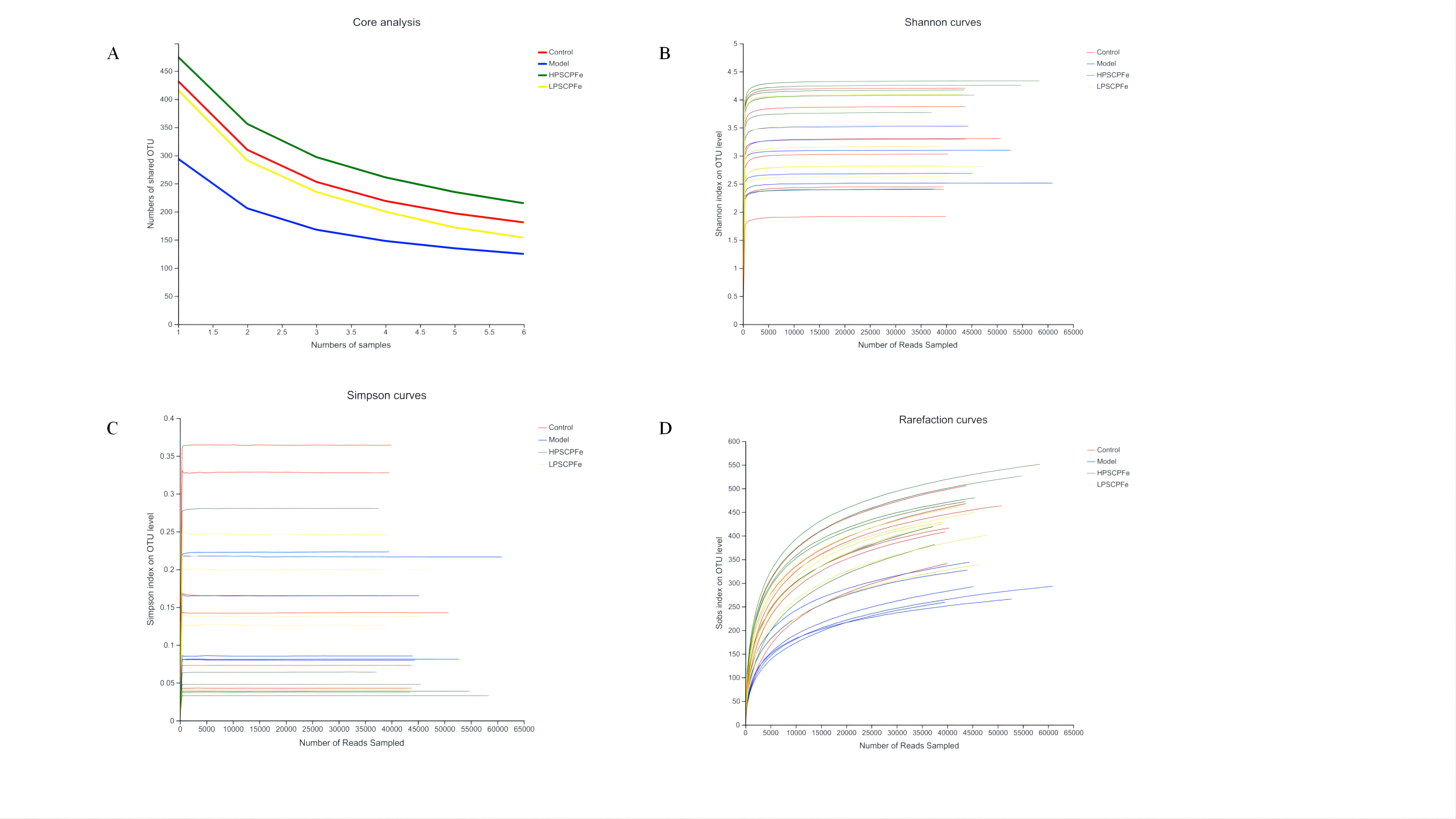
SUPPLEMENTARY FIGURE 1** The curves of core (A) , Shannon (B), Simpson (C) and rarefaction (D).
